# High-Density and Resolution Epicardial Mapping of the Atria: Translational Research with Clinical Impact

**DOI:** 10.3390/jcm13216386

**Published:** 2024-10-25

**Authors:** Ziliang Ye, Yifan Jia, Mathijs S. van Schie, Paul Knops, Vehpi Yildirim, Yannick J. H. J. Taverne, Natasja M. S. de Groot

**Affiliations:** 1 Department of Cardiology, Erasmus Medical Center, 3015 GD Rotterdam, The Netherlands; z.ye@erasmusmc.nl (Z.Y.); y.jia@erasmusmc.nl (Y.J.); m.vanschie@erasmusmc.nl (M.S.v.S.); p.knops@erasmusmc.nl (P.K.); v.yildirim@erasmusmc.nl (V.Y.); 2 Department of Cardiothoracic surgery, Erasmus Medical Center, 3015 GD Rotterdam, The Netherlands; y.j.h.j.taverne@erasmusmc.nl; 3 Department of Microelectronics, Signal Processing Systems, Faculty of Electrical Engineering, Mathematics and Computer Sciences, Delft University of Technology, 2628 CD Delft, The Netherlands

**Keywords:** high-density and resolution epicardial mapping, atria, arrhythmogenic substrate, atrial fibrillation

## Abstract

The electrical arrhythmogenic substrate underlying the most common cardiac arrhythmia atrial fibrillation (AF) may consist of conduction disorders, low-voltage areas, or fractionated potentials. High-density and resolution epicardial mapping (HDREM) approaches have been introduced to quantify and visualize electrophysiological properties of the atria. These approaches are essential for obtaining innovative insights into arrhythmogenic substrates and identifying novel targets for therapy. The aim of this review is to summarize and discuss the (1) contribution of HDREM studies to the knowledge on atrial arrhythmogenesis and (2) future applications of HDREM of atria in daily clinical practice.

## 1. Introduction

**Figure 1:**
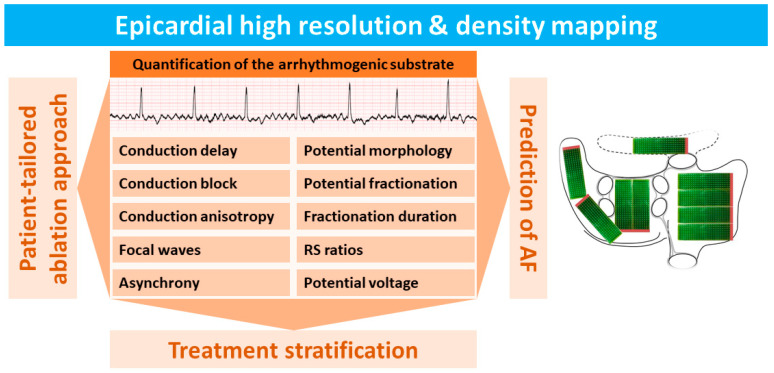
Summary of electrophysiological parameters used for quantification of the electrical arrhythmogenic substrate measured by using high-density and high-resolution epicardial mapping (HDREM) techniques.

In the past decades, high-density and resolution epicardial mapping [1,2,3] (HDREM) studies of the atria have significantly contributed to our knowledge of arrhythmogenesis, particularly of the most common arrhythmia, atrial fibrillation (AF) [4,5]. To date, most HDREM studies of the atria have been performed during open chest surgery. However, the epicardium is not only accessible during open chest surgery, but nowadays also more easily during minimally invasive and video-assisted thoracoscopic surgery [6,7,8]. In addition to this, combined surgical percutaneous ablation strategies for AF are emerging [9,10]. Consequently, epicardial mapping becomes more “common practice” and thus increasingly important. Figure 1 summarizes the electrophysiological parameters recorded in HDREM studies and highlights their role in investigating the arrhythmogenic substrate underlying AF.

An epicardial mapping approach has several advantages over an endocardial mapping approach. First, the surgeon can see the position and orientation of the electrode array in relation to anatomical structures. Second, stable contact between the electrodes and the atrial wall is ensured by directly pressing the electrode array slightly on the epicardial surface. Third, the large opening created by median sternotomy enables introduction of relatively large mapping devices containing a large number of electrodes compared to the currently available endovascular mapping devices. This wide access also facilitates high-density recordings of contact electrograms from numerous sites simultaneously. Fourth, specific areas such as Bachmann’s bundle [11] (BB), which cannot be accessed via the endocardial approach, can easily be mapped. Fifth, simultaneous mapping of the epicardium and the opposite endocardium at a high-density scale can be performed using a small incision in the atrial wall.

In this review, we discuss the main contribution of HDREM of the atria to our knowledge of arrhythmogenesis and the impact of this technology on clinical practice.

## 2. History of Epicardial Mapping

### 2.1. Reentry as the Underlying Mechanism of AF

The first epicardial mapping of the atria was performed in dogs during sinus rhythm by Boineau et al. [12] in 1978. They used 2 electrode templates consisting of a total of 96 bipolar electrodes that were sutured to the atria (electrode diameter 0.2 mm, inter-electrode distance 1 mm, 3 mm between each bipolar pair). Later in 1991, Cox et al. [13] were the first to create an epicardial isochronal map of one sinus rhythm beat activating both the right and left atrium in a human. In addition, Cox et al. [13] performed the first epicardial mapping study of the atria during AF in patients with Wolff–Parkinson–White syndrome undergoing cardiac surgery for interruption of their accessory atrioventricular pathway. AF was induced by burst-pacing and both the right (RA) and left atria (LA) were mapped using epicardial electrode arrays (160 bipolar electrodes, inter-electrode distance 5–10 mm). Their mapping data supported reentry as the underlying mechanism of AF. Non-uniform conduction around areas of bi-directional CB resulted in multiple discrete wavefronts that were observed in all patients. Reentry mostly occurred around anatomical obstacles such as the pulmonary and caval veins, but it also occurred without the involvement of anatomical obstacles. Functional CB was observed in the atria and was sometimes associated with specific atrial structures such as the crista terminalis. In the LA, reentrant circuits were more difficult to document and seemed to occur more transiently. However, in this first epicardial mapping study of AF in humans, only *single* maps were presented, and beat-to-beat changes in patterns of activation were not analyzed.

#### Patterns of Activation

In 1994, HDREM of electrically *induced AF* was performed by Konings et al. [14] in 25 patients with Wolff–Parkinson–White syndrome. During cardiac surgery, the free wall of the RA was mapped with a spoon-shaped electrode array containing 244 unipolar electrodes (diameter 4 cm, inter-electrode distance 2.25 mm). The pattern of activation of the RA free wall showed a high intra- and inter-individual variation and ranged from single planar wavefronts propagating with only small areas of conduction delay to completely disorganized activation by multiple wavefronts propagating in different directions separated by long lines of CB. The degree of the organization of the activation patterns was related to the AF cycle length; long cycle lengths were associated with a higher degree of organization, whereas short cycle lengths were associated with more disorganized activation patterns.

Based on the complexity in the patterns of activation, AF was divided into three different types. During type I AF, the RA free wall was most often activated by single broad wavefronts and only small areas of slow conduction or CB occurred. These areas of conduction disorders did not affect the main course of the large fibrillation waves. During type II AF, there were either areas of local conduction delay affecting propagation of one large wavefront, or two separate wavefronts were present. During type III AF, multiple fibrillation waves were separated by functional lines of intra-atrial CB. The incidence of type I, II, and III AF in this study population of 25 patients was, respectively, 40%, 32%, and 28%. During these three distinct types of AF, there were significant differences in the average conduction velocity; type I AF was associated with the highest conduction velocity (61 ± 6 cm/s) and type III AF with the lowest conduction velocity (38 ± 10 cm/s). However, by using the “longitudinal” and “transverse” conduction velocity, Houben et al. [15] demonstrated that there was no difference in anisotropy ratio among the three types of AF. Even taking the highest degree of directional differences in conduction, the anisotropy ratio during AF still was not more than 1.80 ± 0.39. The initial mapping studies analyzing patterns of activation at the RA in patients with *(longstanding) persistent AF* were mainly performed during cardiac surgery for valvular heart disease [16,17].

Other mapping studies of chronic AF, as named at that time, focused on comparing patterns of activation in the RA and LA, which were mapped sequentially [18,19]. Surprisingly, in most patients, activation of the LA was more organized compared to RA. In three patients, organized and disorganized activation occurred alternately in the RA. However, since the mapping resolution of these studies was rather low (only 30 unipolar or 24 bipolar electrodes), conduction abnormalities occurring in small areas could easily have been missed. More recent studies performed by De Groot et al. used dedicated HDREM electrode arrays containing either 64 (8 × 8 electrodes, 1.10 cm^2^, inter-electrode distance 1 mm) [20], 128 (8 × 16 electrodes, inter-electrode distance 2 mm), or 192 unipolar electrodes [21] (8 × 24 electrodes, electrode diameter 0.45 mm, inter-electrode distance 2 mm). Other studies of Kalman et al. [22] used custom-made electrode arrays of 128 electrodes (inter-electrode distance 2.5 mm). Electrode arrays have also more recently been used for simultaneous endo-epicardial mapping of AF [23]. Due to the emergence of “hybrid” ablation procedures, epicardial access becomes more easily achievable. Therefore, it is expected that the number of studies involving HDREM will increase considerably in the near future.

## 3. Historical Insights into AF Mechanisms

Konings et al. [14] observed a considerable number of focal patterns of activation at the RA epicardium during *acutely induced AF* in patients with Wolff–Parkinson–White syndrome. They described these focal waves as “new fibrillation waves” emerging in the middle of the mapping area, which could not be explained by fibrillation waves propagating in the epicardial plane. Holm et al. [17] also observed repetitive focal activation patterns in the RA during *persistent AF*, which was consistently located at the RA appendage.

Many years later, high-resolution and density epicardial fibrillation “wave” maps of the RA and LA obtained from patients with Wollf–Parkinson–White syndrome in whom AF was acutely induced and patients with coronary artery- and valvular heart disease who had longstanding persistent AF (LSPAF) were compared [24]. A wave map shows the area activated by each individual fibrillation wave in color-code according to the moment of entrance into the mapping area. Utilizing these wave maps to count the number of fibrillation waves, it was demonstrated that persistence of AF was associated with an increase in the number of fibrillation waves (LSPAF: 4.5/cm^2^ versus acutely induced AF: 2.3/cm^2^, *p* < 0.001). In addition, patients with long-standing persistent AF (LSPAF) had more lines of CB (CB) (21.1 [16.6–27.5] vs. 6.1 [1.3–13.8]%, *p* < 0.001), which were also longer (total length: 21.1 [19.1–23.1] vs. 3.4 [0.3–8.8] mm/cm^2^, *p* < 0.001). CB lines were defined as inter-electrode conduction times of >12 ms, representing a lower limit of 19 cm/s conduction velocity.

Recently, Van Schie et al. [25] demonstrated that increased AF complexity was associated with increased spatiotemporal variability in local conduction velocity vectors, local conduction heterogeneity, and anisotropy ratio. Interestingly, as shown by De Groot et al. [26] AF persistence was also associated with a higher frequency of epicardial breakthrough (“focal”) waves, as the prevalence of focal fibrillation waves in the RA during LSPAF was almost four times higher than it was during acute AF (median, 0.46 versus 0.12 per cycle per 1 cm^2^; *p* < 0.0001).

The observation that focal waves are associated with AF persistence raised the question whether focal waves can be considered as drivers of AF, and, if so, whether they can be targets for ablation therapy in patients in whom AF persists despite isolation of the pulmonary veins. However, the focal waves occurred diffusely throughout the atria, and predilection sites did not occur. Focal waves often arose as solitary events, and they rarely occurred repetitively.

Potentials recorded at the origin of the focal wave usually had clear R-waves preceding a negative deflection [26], indicating propagation of a wavefront towards the recording electrode. The coupling interval of the focal waves was <11 ms shorter than the average AF cycle length, indicating that these focal waves cannot be regarded as drivers of the fibrillatory process.

The occurrence of non-repetitive focal waves during AF was also reported by Lee et al. [27] They performed epicardial mapping (128 electrodes, inter-electrode distance of 2.5 mm) of various atrial regions—including the posterior LA wall, LA appendage, RA appendage, and right superior pulmonary vein–LA junction—in patients with LSPAF and coronary artery or valvular heart disease. None of the focal activation patterns lasted longer than two consecutive beats. Likewise, Walters et al. [3] performed in 10 patients epicardial mapping of the posterior LA wall and the RA free wall and found that the median number of consecutive focal waves was only 2 (IQR: 2–3), whereas the longest series of consecutive focal waves was 5. In a large cohort of 71 patients with different types of AF, van Staveren et al. [28] demonstrated that sites harboring focal waves were not the sites with the highest dominant frequency and highest degree of fractionated electrograms according to existing criteria for AF ablation. Hence, data from these translational mapping studies in humans did not support the concept that focal waves are the drivers of the fibrillatory process and that focal waves are suitable target sites for ablation therapy.

### The Double-Layer Hypothesis

**Figure 2:**
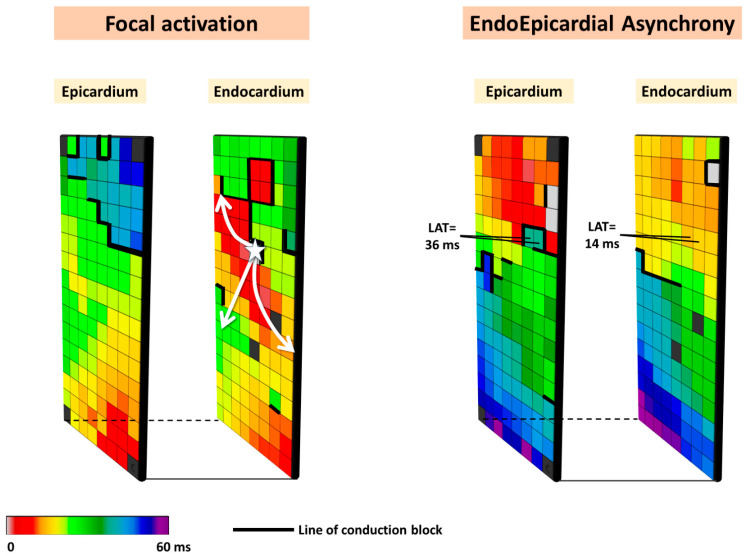
Simultaneously recorded epicardial and opposite endocardial activation maps. Local activation times are color-coded, and lines of CB are indicated by thick black lines. The left panel shows an example of focal activation (white star) on the endocardial layer, while the right panel shows an example of endo-epicardial asynchrony.

Based on the observation that focal waves were associated with AF persistence, the so-called double-layer hypothesis was introduced. In this hypothesis, the substrate of persistent AF consists of electrically dissociated muscle bundles and the mechanism of persistent AF is multiple endo-epicardial breakthroughs caused by transmural propagation of fibrillation waves from one layer to the other (right panel of Figure 2). These transmurally propagating fibrillation waves provide a constant source of new fibrillation waves for the opposite layer. However, this hypothesis requires that areas of electrical asynchrony between the endo- and epicardium are present, as otherwise, transmurally propagating waves could not excite the opposite layer.

## 4. Simultaneous Endo-Epicardial Mapping during AF

In 1993, Schuessler et al. [29] performed simultaneous epicardial and endocardial mapping of isolated RA in canines during SR, electrical stimulation, and induced tachyarrhythmias. During SR, endocardial and epicardial activation occurred approximately simultaneously, as only small differences between endocardial and epicardial activation times (<1 ms) were found. Larger differences in endo- and epicardial activation times were mainly found at pectinate muscles and sites with transmural differences in fiber orientation; these differences increased during premature stimulation and induced AF. Interestingly, a focal activation pattern was observed, which was caused by a reentrant circuit in free-running muscle bundles connecting the epicardium and endocardium. The earliest activated sites giving rise to focal activation on either the endo- or epicardium were separated by 15 mm [29].

To prove that electrical asynchrony also exists in the in vivo human heart, De Groot et al. [23] performed, for the first time, simultaneous endo-epicardial mapping of the RA wall during AF in patients undergoing cardiac surgery and demonstrated frequent electrical asynchrony between the endo- and epicardial layers (up to 55.9% of the recording time). There were not only large differences in opposite local activation times but also differences in activation direction. Electrical asynchrony was more pronounced in patients with LSPAF compared to paroxysmal AF, indicating that electrical asynchrony across the atrial wall plays a key role in AF persistence. Due to the risk of air embolism, simultaneously mapping of the endo- and epicardium was limited to the RA. If extensive areas of asynchrony perpetuating the fibrillatory process are present in the atria, the *double-layer hypothesis* offers an explanation as to why patients have AF recurrences despite complete isolation of the pulmonary veins.

## 5. AF Potential Morphology

**Figure 3:**
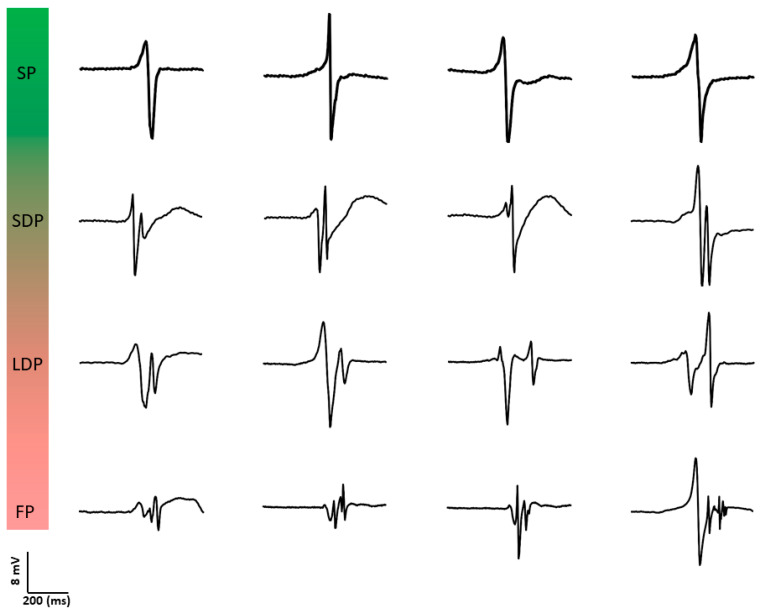
Examples of different types of unipolar potential morphologies. SP: single potential; SDP: short double potential; LDP: long double potential; FP: fractionated potential.

Konings et al. [30] were also the first to investigate the relation between unipolar potential morphology and spatial activation patterns during AF. They classified unipolar fibrillation potentials as SP, short double (SDP, two deflections, <10 ms apart), long double (LDP, two deflections, 10–50 ms apart), and fragmented potentials (FP, >2 deflections within 50 ms). Figure 3 provides typical examples of different unipolar potential morphologies. During induced AF, 77 ± 12% of potentials were SPs, 7 ± 3% SDPs, 10 ± 7% LDPs and 6 ± 4% FPs. SPs were an indicator for rapid uniform conduction (positive predictive value of 0.96). This type of conduction was present in most wavefronts (79 ± 11%); in 94 ± 4% of potentials within these wavefronts, SPs were observed. LDPs were specific for long lines of functional CB, whereas FPs were observed both during pivoting of fibrillation waves and during slowing of conduction. The complexity in patterns of activation was associated with unipolar potential morphology. Hence, the proportion of LDPs and FPs was highest during type III AF.

The morphology of single potentials is represented by the relative sizes of their positive (R-wave) and negative (S-wave) components (R/S ratio). Houben et al. [15] were the first to examine the morphology of SPs during acutely induced AF at the RA. In total, 413,031 unipolar fibrillation potentials were analyzed. Although there was a large intra- and inter-individual variation, a clear predominance of S-waves was found. The S-wave predominance was less pronounced during the more complex type of AF (type III) and could not be attributed to wavefront curvature or anisotropy. It was therefore hypothesized that a tilted transmural stance of the wavefront, resulting in an epicardial lead with constant epi-to-endocardial activation, could explain the S-wave predominance.

Based on the observations of Konings et al. [30], Nademanee et al. [31] developed a substrate-based AF ablation approach targeting complex fractionated atrial electrograms (CFAEs). This ablation approach has now been abandoned, which is not surprising given the significant variability in recording methodologies and definitions of CFAE used. Currently, there are at least 27 different definitions [32] used for identification of CFAEs, which leads to considerable inconsistency in treatment strategies. The CFAE approach is further complicated by the different recording techniques employed [33], including variable electrode sizes, inter-electrode distances, and filter settings. The variability in the methodology of CFAE detection explains substantial differences in ablation outcomes across different centers and operators, highlighting the need for a more standardized and reliable approach for CFAE mapping. We still believe that unipolar potentials are indicators of the AF-related arrhythmogenic substrate. However, to understand which features of potential morphologies are more closely related to AF development, thorough knowledge of the variation in these features during normal heart rhythm is essential. For this purpose, HDREM is used to quantify features of electrogram morphology, including potential voltages, degree of fractionation, duration of fractionation, and R/S ratios of single potentials.

## 6. Staging of AF by Quantifying Electropathology

The observation that AF persistence was associated with a larger number of fibrillation waves and more and longer lines of CB suggested that the arrhythmogenic substrate, and thus AF, can be “staged” by quantifying features of activation patterns and potential morphology. Such a diagnostic tool to stage AF could be used in clinical practice to provide patient-tailored therapy. Once an invasive “gold standard” diagnostic tool is developed and the exact AF-related electropathology has been identified, it can be used to construct less invasive and ultimately non-invasive diagnostic AF staging tools.

**Figure 4:**
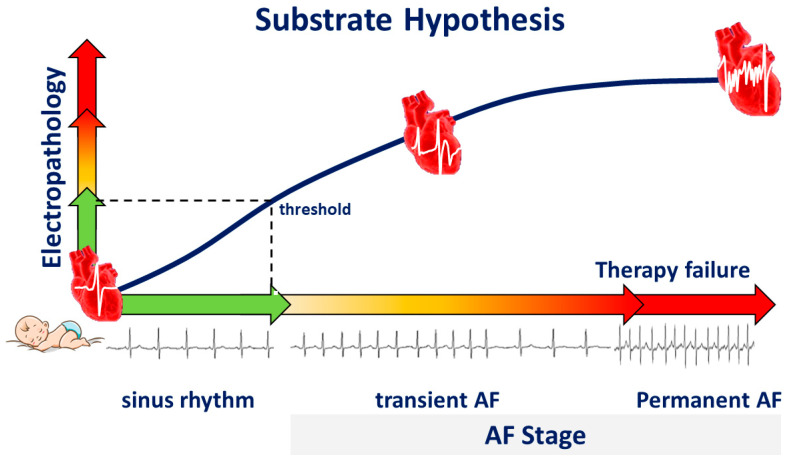
Substrate hypothesis. During life, electropathology progresses, and the heart rhythm changes from sinus rhythm to transient atrial fibrillation (AF), and finally to permanent AF, a condition frequently associated with therapy failure.

We hypothesize that during life, damage to atrial tissue by, e.g., cardiovascular diseases and co-morbidities causes “electropathology” manifested as pro-arrhythmogenic changes in patterns of activation and potential morphology. This tissue damage accumulates until a certain threshold of electropathology is reached and arrhythmias arise (Figure 4). From that moment on, additional electropathology contributes to perpetuation of the arrhythmia. Despite extensive research, the exact features of the AF-related arrhythmogenic substrate are unknown. To unravel the AF-related arrhythmogenic substrate, several steps are necessary, including understanding of (1) intra-atrial variation in activation patterns and potential morphology during normal heart rhythm, (2) the impact of clinical characteristics on atrial electrophysiology, and (3) differences in atrial electrophysiology between patients with and without AF. For this purpose, extensive mapping studies of the atria during sinus rhythm (SR) have been performed in a variety of patient populations.

## 7. Epicardial Sinus Rhythm Mapping

The first detailed activation time mapping of the atria was performed in dogs during sinus rhythm by Boineau et al. [12] in 1978. Later, Cox et al. [13] were the first to create an epicardial isochronal map of one SR beat activating both the right and left atrium in a human. In 2018, Mouws et al. [34] were the first to investigate high-resolution atrial excitation patterns during normal SR in 253 patients with ischemic heart disease or ischemic valvular heart disease. As expected, in the vast majority (92%) of the patients, SR activations originated from the superior intercaval region of the RA. This study also highlighted the complex nature of BB activation, which exhibited various patterns, including right-to-left activation (64%), an expanding wavefront emerging from the central part (7%) of BB, by multiple wavefronts entering BB from different directions in the remaining patients. Furthermore, in 42% of the patients, the LA was activated by wavefronts propagating via BB towards the left atrioventricular groove. In a small number of patients, the SR wavefront propagated from the septum or coronary sinus across the pulmonary vein area (PVA, 3%) towards the LA appendage. However, in most patients (52%), there was a combination of both activation patterns. These findings provide insights into the variable patterns of atrial activation during normal SR. Additionally, it was found that the activation pattern of BB is particularly affected by valvular heart disease, which may explain why patients with valvular heart disease have a higher risk of developing AF.

### 7.1. Influence of Prior AF Episodes on Atrial Excitation

Mouws et al. [34] also demonstrated that patients with a history of AF were characterized by a longer total atrial excitation time during SR compared to patients without AF (136 ± 20 ms vs. 114 ± 17 ms, *p* < 0.001), which was mainly caused by longer total activation times of the right atrium and Bachmann’s bundle (73 ± 13 ms vs. 67 ± 14 ms, *p* = 0.018 and 106 ± 20 ms vs. 87 ± 16 ms, *p* < 0.001, respectively). The presence of extensive conduction disorders at these areas resulted in alternative routes for activation of Bachmann’s bundle and the left atrioventricular groove, as Bachmann’s bundle could be activated via one wavefront from right to left, from the central part or via multiple wavefronts [35]. The left atrioventricular groove was then activated via either Bachmann’s bundle, the pulmonary vein area, or via both routes, depending on which route had the shortest interatrial excitation time. However, Mouws et al. [34,36] also demonstrated that excitation of the left atrioventricular groove via only the pulmonary vein area was considerably slower than via Bachmann’s bundle (90 ± 18 ms vs. 101 ± 20 ms, *p* < 0.001), and increased the risk of AF. Regional differences in local conduction velocities (CV) were investigated by Van Schie et al. [37] In 412 patients with ischemic and/or valvular heart disease, they found that CV was highest at the PVA [94.3 (83.4–101.0 cm/s)], followed by the LA [90.2 (82.2–97.0 cm/s)] and the RA [89.1 (83.3–94.3 cm/s)]; CV was lowest at BB [86.9 (77.3–96.4 cm/s)]. In patients with paroxysmal AF, local CV at BB was lower compared to those without prior AF episodes (79.1 [72.2–91.2] vs. 88.3 [79.3–97.2] cm/s, *p* < 0.001). In a subsequent case–control study, Heida et al. [38] confirmed there was a reduction in CV at Bachmann’s bundle in patients with a history of AF (79 ± 12 vs. 88 ± 11 cm/s, *p* = 0.02).

The impact of underlying heart disease, including coronary artery, valvular heart, and congenital heart disease on conduction heterogeneity was explored in a study involving 447 patients who underwent epicardial mapping during SR [39]. Interestingly, the underlying heart disease had no effect on the proportion and severity of conduction disorders. However, prior AF episodes were associated with slowing of conduction in both atria and more conduction disorders at BB. In patients with AF, there are no differences in biatrial conduction characteristics between the supervulnerable period after electrical cardioversion and long-term SR in AF patients [40].

**Figure 5:**
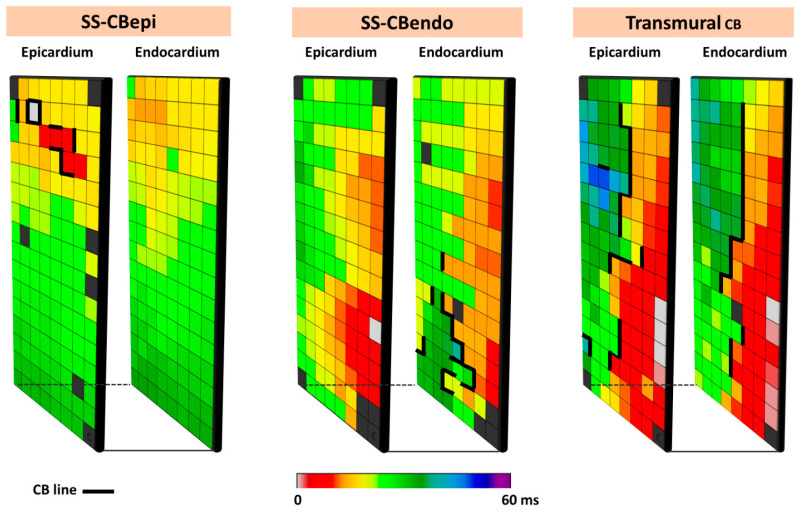
Different types of CB observed during endo-epicardial mapping. The left, middle, and right panels show, respectively, single-sided epicardial CB, endocardial single-sided CB, and transmural CB. SS-CB_endo_: endocardial single-sided CB; SS-CB_epi_: epicardial single-sided CB; Transmural CB: transmural CB.

The complexity of CB even during SR is demonstrated by simultaneous endo-epicardial mapping studies of the RA, which revealed that lines of CB can be located at either the endo- or epicardium only (single-sided CB) or at both layers (transmural CB) [41]. Figure 5 illustrates different types of CBs observed during simultaneous endo-epicardial mapping.

### 7.2. Focal Patterns of Activation During SR

If focal waves due to transmurally propagating fibrillation waves are the result of electrically dissociated muscle bundles caused by structural changes in the atrial wall, it is most likely that focal patterns of activation may also appear during SR. In search of these focal patterns of activation during SR, Mouws et al. [42] performed epicardial mapping studies in 381 patients with ischemic or valvular heart disease. In total, 218 focal waves at various locations were found in 168 patients. These locations included mainly the RA (N = 105, 48%) but also the left atrioventricular groove (N = 67, 31%), BB (N = 27, 12%) and the PVA (N = 19, 9%). Focal waves occurred more frequently in patients with ischemic heart disease (N = 114, 49%) compared to patients with ischemic and/or valvular heart disease (N = 26, 17%). In this study [42], the morphology of SR potentials at the origin of the focal wave was also examined. These potentials were most often double or fractionated potentials (N = 137, 63%) and mainly occurred at focal patterns of activation arising from the RA and BB. In case of SPs, the majority (N = 71, 88%) contained a clear R-wave. These features of potential morphology indicate that there are indeed waves propagating from deep layers within the atrial wall towards the recording electrode.

As focal waves are present during SR, it indicates that endo-epicardial asynchrony must also be present during SR. Kharbanda et al. [43] conducted simultaneous endo-epicardial mapping of the human RA and indeed demonstrated endo-epicardial asynchrony even up to 61 ms in the thin inferior RA and up to 84 ms in the thick superior RA. Areas of endo-epicardial asynchrony are also more prevalent during SR in patients with persistent AF compared with patients without history of AF and can be more accurately detected by utilizing unipolar electrograms than bipolar electrograms.

### 7.3. Unipolar Potential Morphology

The value of unipolar potential morphology has been discussed for many years as a possible indicator of the electrical arrhythmogenic substrate. Potentials consisting of RS waves with high amplitudes reflect areas of fast conduction with conduction along the longitudinal axis of myocardial fibers, while in areas of slower conduction, unipolar potential amplitudes are smaller [44]. Low-voltage areas are assumed to be indicators of structurally remodeled tissue containing fibrotic areas, though study outcomes are controversial [44,45]. Asynchronous activation of (groups of) cardiomyocytes gives rise to multiple positive and negative deflections, which is known as fractionation [44,46].

### 7.4. Unipolar Single Potential Morphology

SPs are defined as potentials consisting of a single negative deflection, and the preceding and following wave are called, respectively, the positive R- and negative S-wave. In patients with coronary artery disease without history of arrhythmias, the majority of unipolar potentials recorded during normal SR consisted of one single deflection (81.4 [74.9–85.1]%) [40]. Even in patients with mitral valve disease and paroxysmal AF, the majority of recorded potentials still consists of SPs (81.0 [75.5–87.0]%) [47,48]. The morphology of SPs is represented by the relative positive (R-wave) and negative (S-wave) components (R/S ratio).

As described above, Houben et al. [15] were the first to examine the morphology of SPs during acutely induced AF at the RA. They proposed that the thin epicardial layer of atrial myocardium plays an important role in propagation of fibrillation waves. Simultaneous endo-epicardial mapping of the RA during SR, however, demonstrated that opposite epicardial and endocardial electrograms both have S-predominance [49], suggesting that predominant epicardial-to-endocardial activation during SR is not the cause of S-predominance.

Differences in relative R- and S-wave amplitude ratios of SPs were further studied in a cohort of 67 patients with mitral valve disease [47]. In all patients, there was a clear predominance of S-waves at BB and the RA. Comparing patients without AF and with PAF, SP morphology differences were most prominent at BB. Patients with PAF had lower amplitudes, more R-wave predominance, and slower wavefront propagation. The lower amplitude was mainly determined by a decrease in S-wave amplitude, which is observed in damaged tissue. SPs were also recorded from the origin of epicardial focal waves during SR; an R-wave was observed in 88% of all epicardial focal waves, as opposed to in only 21% of sinus node breakthrough waves [42].

### 7.5. Double and Fractionated Potentials

Ye et al. [50] described the distribution and the proportion of different types of atrial unipolar potential morphologies during SR. The study involved 189 patients undergoing coronary artery bypass graft surgery, preceded by epicardial mapping of the RA, BB, PVA, and LA. Various types of potential morphologies, including SPs, SDPs, LDPs, and FPs, were distributed across the different regions of the atria. The highest proportions of LDP and FP were mainly recorded at the RA and Bachmann’s bundle, with fractionation at Bachmann’s bundle also having the longest durations. The amount of CB was correlated with the proportion of LDPs and FPs, and duration of FPs. In a cohort of patients with mitral valve disease and paroxysmal AF, it was demonstrated that more unipolar FPs were present in the pulmonary vein area compared to patients without a history of AF [48]. However, LDPs and FPs were detected throughout the atria in patients without atrial tachyarrhythmias. This finding suggests that unipolar potential fractionation can also be physiological in nature, due to, for example, tissue discontinuities caused by anatomical structures such as capillaries. Interestingly, by performing simultaneous endo-epicardial mapping, Zhang et al. [51] demonstrated that LDPs and FPs are also indicators of areas of electrical asynchrony between the endo- and epicardial layers. The inability to discriminate physiological fractionations from pathological ones provides a possible explanation for the limited success of ablation strategies targeting CFAE.

### 7.6. Potential Voltages

It is generally assumed that potentials with low peak-to-peak amplitudes (voltages) are indicators of sites of structurally remodeled tissue and have therefore become targets for AF ablation [52]. For this reason, several HDREM studies have also focused on investigating potential voltages. However, the use of potential voltage as an indicator for arrhythmogenic tissue critically relies on the used voltage mapping technique. Mapping studies used several voltage mapping approaches including unipolar, bipolar, omnipolar, Laplacian, or multipolar voltage techniques, which all have their advantages and disadvantages [32]. There is no standardized definition for low-voltage areas and a clear threshold has never been histologically validated [53]. Also, low-voltage areas defined with unipolar potentials may differ considerably from low-voltage areas defined with bipolar or omnipolar voltages [54]. Simultaneous endo-epicardial mapping studies have demonstrated that low-voltage areas are not always transmural but can be present in either the endocardium or epicardium [55,56]. Interestingly, low-voltage, fractionated potentials were not only recorded in areas with slowing of conduction or CB [54,55], indicating that not all FPs are indicators of an arrhythmogenic substrate, but could simply be the result of structure of the atrial wall.

In order to fully exploit unipolar potential voltages for detecting AF vulnerability, more insights into the distribution of unipolar potential voltages during sinus rhythm were required by van Schie et al. [48]. From 67 patients with mitral valve disease, high-resolution unipolar SR voltage fingerprints to identify low-voltage areas were constructed and compared between patients with and without AF episodes. They demonstrated that there was no correlation between unipolar potential voltages and CV, although lower potential voltages were recorded in areas of conduction slowing and surrounding areas of CB.

In addition, unipolar potential voltages decreased in potentials with an increasing number of deflections. Even during SR, marked inter-individual and regional variation in potential voltages was found. However, patients with paroxysmal AF had lower unipolar potential voltages at RA, Bachmann’s bundle, and PVA, and more low-voltage areas at Bachmann’s bundle compared to those without AF. This difference was mainly determined by the magnitude of the S-waves, which was particularly lower at BB [47]. In 189 patients undergoing epicardial mapping of the atria during SR, Ye et al. [50] found a significant negative correlation between unipolar potential voltages and CB in the RA, BB, and the PVA. These observations suggest that unipolar potential voltages can be used to assess the severity of atrial conduction abnormalities.

## 8. Electrical Signal Fingerprinting

**Figure 6:**
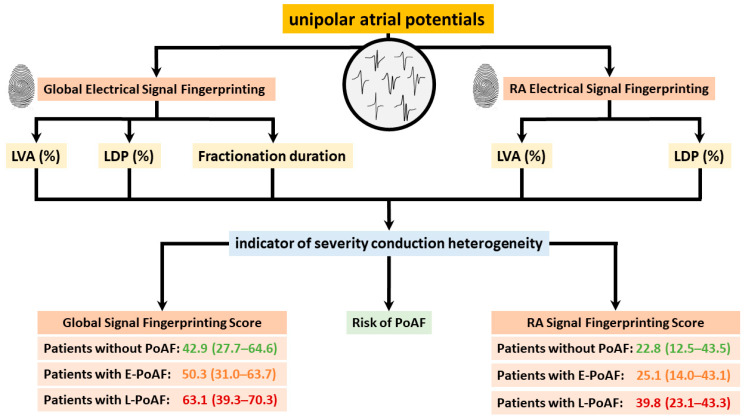
Electrophysiological parameters of global and right atrial electrical signal fingerprinting, along with their application in predicting postoperative atrial fibrillation, are illustrated. LVA: low-voltage area; LDP: long double potential; PoAF: postoperative atrial fibrillation.

In 2021, Ye et al. [50] first introduced the concept of electrical signal fingerprinting in a large cohort of patients without atrial tachyarrhythmias. The electrical signal fingerprint uses features of unipolar potential morphology to predict the severity of atrial conduction heterogeneity in the epicardial layer. The fingerprint was then tested in 235 patients undergoing coronary artery bypass grafting; the electrical signal fingerprint score had a significant predictive value of conduction heterogeneity (entire atrium: C-index: 0.92 (0.89, 0.95)) [57]. Moreover, patients who developed postoperative AF had a markedly higher electrical signal fingerprint score compared to patients without postoperative AF. This observation suggests that the electrical signal fingerprint score could serve as a novel diagnostic tool for prediction of postoperative AF. The electrophysiological parameters of the electrical signal fingerprint and their further utilization in identifying patients at higher risk of postoperative AF are illustrated in Figure 6. Zhang et al. [41] discovered that unipolar potential morphologies were also useful in identifying areas of transmural CB, which could accurately be predicted by combining unipolar potential morphology parameters, including potential voltages, fractionation, and fractionation duration (area under the curve (AUC) = 0.91).

Electrical signal fingerprints can also be used to identify areas of endo-epicardial asynchrony (EEA) during SR. Zhang et al. [51] performed simultaneous endo-epicardial mapping in a cohort of 86 participants and revealed that endocardial low-voltage areas and fractionation duration of double potentials (DPs) serve as significant predictors for the degree of EEA, achieving an AUC of 0.913. This high predictive value underscores the potential utility of these electrophysiological parameters in the assessment of EEA. Moreover, endo- and epi-asynchrony fingerprinting scores (AFS) were introduced to use either endocardial or epicardial mapping to identify EEAs. Both methods had a strong predictive value for the prediction of EEA, with AUCs of 0.901 and 0.830, respectively. These findings highlight the efficacy of AFS as a less-invasive diagnostic tool compared to simultaneous endo- and epicardial mapping to quantify the severity of EEA, which is potentially an indicator for AF vulnerability.

## 9. Impact of Risk Factors on Atrial Epicardial Electrophysiology

In the past decade, epicardial mapping studies have been performed in large patient cohorts undergoing cardiac surgery to study the impact of risk factors on atrial electrophysiology. These risk factors include ageing, sex, and obesity.

### 9.1. Ageing

The impact of age on atrial electrophysiological properties was studied in 216 patients undergoing elective coronary artery bypass surgery [58]. With aging, slowing in conduction and CB occurred during SR throughout both the RA and LA. Conduction abnormalities were particularly more prevalent at the RA and BB. Furthermore, this was the first study demonstrating that at BB, with advancing age, slowing of conduction in transverse direction occurred to a greater extent compared to the conduction slowing in longitudinal direction, reflecting enhanced lateral uncoupling.

Ye et al. [59] further explored the relationship between ageing and epicardial unipolar atrial potential morphology. Advanced age, which was defined as age ≥ 60 years, was associated with a decrease in SPs, an increase in SDPs, LDPs, and FPs. Even after adjusting for potential confounding factors, the impact of age on these four different types of potential morphology remained significant. Both of the above-mentioned studies were conducted during SR, and there are currently no data about the impact of age on the fibrillatory process.

### 9.2. Obesity

The relation between obesity and AF has been demonstrated by several clinical studies, although the pathophysiology of obesity-related AF remains largely unknown. The impact of obesity on atrial electrophysiology was investigated by Schram-Serban et al. [60], who performed epicardial mapping studies in 106 matched obese patients and non-obese patients. None of these patients had a history of AF. Compared to non-obese patients, obese patients had more areas of conduction delay, CB, and longer continuous conduction delay and CB lines. These differences were most pronounced at BB. Furthermore, in a subsequent study, Schram-Serban et al. [61] demonstrated that obese patients also had lower unipolar potential voltages, particularly at BB and the LA. Furthermore, the number of low-voltage areas positively correlated with incidences of CB, while BMI and the number of low-voltage areas were independent predictors for the incidence of early postoperative AF. This study suggests that obesity may predispose to an overall decrease in atrial potential voltages and a higher number of low-voltage areas, and that Bachmann’s bundle is a predilection site for low-voltage areas within the atria of obese patients. The next step in further understanding the obesity-related electrical substrate underlying AF is to investigate the electrophysiological differences between obese and non-obese patients with a history of AF to investigate whether obesity further exacerbates atrial remodeling in AF patients.

### 9.3. Sex Differences

There is increasing awareness of sex-specific differences in epidemiology, pathophysiology, and therapy outcomes of AF. To fully understand these differences, knowledge on sex differences in atrial electrophysiological properties during normal heart rhythm is essential. However, data on this topic are scarce. Therefore, Veen et al. [62] used epicardial mapping to investigate the impact of sex on electrophysiological properties of the atria during SR in 53 matched post-menopausal females and males. None of these patients had a history of AF. Sex-related differences in electrophysiology were particularly found at the RA and to a lesser degree at BB. At both the RA and BB, females had significantly lower unipolar potential voltages and conduction velocities. At the RA only, females had more low-voltage areas, more CB, and continuous conduction delay and CB lines, and also longer CB and continuous conduction delay and CB lines. In future studies, it needs to be investigated whether these areas play a role in sex-based differences in vulnerability to atrial tachyarrhythmias such as AF.

## 10. Gaps in Current HDREM Research

At present, HDREM is used by only a small number of researchers and experience with this technology is therefore limited. As recently discussed in detail [32], differences in signal recording and particularly processing techniques may exist and it is unknown how these methodological differences affect reproducibility of study outcomes.

## 11. Clinical Applications of Epicardial Mapping

HDREM can also be applied to guide real-time, patient-specific therapy. Although epicardial mapping is predominantly performed during open chest surgery, it is nowadays also increasingly conducted during minimally invasive procedures. As concomitant surgical AF ablation is advised in patients with paroxysmal or persistent AF and stand-alone surgical or hybrid ablation is considered appropriate in symptomatic patients with persistent AF, the clinical applications of epicardial mapping to guide these procedures is expanding.

Comparable to endocardial pulmonary vein isolations in the electrophysiology (EP) room, HDREM can be used to evaluate continuity of epicardial lesions during arrhythmia surgery and provide feedback on exit and/or entrance block to the surgeon. Secondly, as discussed above, the *Electrical Fingerprint Scores* obtained via epicardial mapping can provide patient-tailored AF therapy. Theoretically, if the score is low, pulmonary vein isolation may be sufficient, and if this score is high, a substrate-based AF ablation approach may be more suitable. Quantification of HDRM parameters derived from epicardial mapping enables identification of the arrhythmogenic substrate with an unparalleled accuracy. Furthermore, by correlating *electrical signal fingerprinting scores* derived from epicardial mapping data with non-invasive electrocardiographic features, it may be possible to develop a more advanced non-invasive diagnostic tool to quantify the severity of conduction inhomogeneity across the entire atrium, thereby identifying individuals at higher risk of developing AF.

Epicardial mapping generates a large amount of electrophysiological data from the atria. The integration of HDREM with artificial intelligence, along with the development of less invasive and non-invasive techniques, is likely to yield novel insights into the AF-related arrhythmogenic substrate and hence unravel novel target sites for therapy. Thus, implementation of novel signal processing technologies in HDREM will be a significant advancement in the future management of AF. However, for HDREM to become implemented in daily clinical practice, a standardized approach for signal recording and processing is mandatory.

## 12. Conclusions

HDREM has emerged as a pivotal approach in advancing our understanding and treatment of atrial arrhythmias, particularly AF. This technology enables visualization and quantification of arrhythmogenic substrates, such as conduction disorders, low-voltage areas, or fractionated potentials. In patients with trigger-driven AF undergoing cardiac surgery, it can be used to verify the completeness of ablation lesions, whereas in patients with substrate-mediated AF, it can be used to identify target sites for ablation. In addition, HDREM can be applied to stage AF, provide patient-tailored therapy, or identify patients at high risk of developing AF. Once the exact features of the AF-related substrate have been identified using HDREM, this knowledge can be used to develop less invasive or non-invasive techniques that have broader applications in daily clinical practice.

## Data Availability

Not applicable.
